# Supplementary material for: Using All-Atom Potentials to Refine RNA Structure Predictions of SARS-CoV-2 Stem Loops
Source: Int J Mol Sci. 2020 Aug 27;21(17):6188. doi: 10.3390/ijms21176188 (PMC7504604; doi:10.3390/ijms21176188)
Supplement: Supplementary file 1 [file ijms-21-06188-s001.pdf]

Supporting Information:

Using All-Atom Potentials to Refine RNA Structure Predictions of SARS-CoV-2 Stem Loops

Christina Bergonzo [1,*] and Andrea L. Szakal[2]

1 Institute for Bioscience and Biotechnology Research, National Institute of Standards and Technology and University of Maryland 9600 Gudelsky Drive, Rockville, MD 20850, USA; christina.bergonzo@nist.gov

2 National Institute of Standards and Technology, 100 Bureau Drive, Gaithersburg, MD, 20899, USA; andrea.szakal@nist.gov

* Correspondence: christina.bergonzo@nist.gov; Tel.: +1 (240) 314-6333

Supporting Figure 1. Time dependent RMSDs to heavy atoms of loop and stem of starting SL2 structures. Top: Simulations starting from NMR ensemble structures. Bottom: Simulations starting from FARFAR2 top structures from clusters 1-10. 3

Supporting Figure 2. Histogram of hydrogen bond distance between N3 and H1 atoms of C-G (NMR: C47-G50, FARFAR2: C50-G53, blue) or N3 and H3 atoms of C-U (NMR: C47-U50, FARFAR2: C50-U54, red) measured for SL2 simulations starting from NMR ensemble structures (top) and simulations starting from FARFAR2 top structures from clusters 1-10 (bottom). 4

Supporting Figure 3. Close up of histogram of hydrogen bond distance between N3 and H1 atoms of C50-G53 (top, blue) or N3 and H3 atoms of C50-U54 (bottom, red) measured for SL2 simulations starting from FARFAR2 top structures from clusters 1-10. This is a close up of the bottom plot of Supporting Figure 2. 4

Supporting Table 1. Number of frames and percentage of FARFAR2-SL2 trajectories sampling central CG/CU base pair 5

Supporting Figure 4. Pucker and chi distributions for SL2 MD simulations starting from: deposited NMR models (NMR MD) (blue), FARFAR2 models which sample the central CG hydrogen bond (FAR CG) (cyan) and FARFAR2 models which sample the central CU hydrogen bond (FAR CU) (red). SL2 numbering scheme from NMR structure is used here for both NMR and FARFAR2 models. 5

Supporting Figure 5. Backbone dihedral average values for SL2 loop residues for the deposited MD models (black) MD simulations starting from: deposited NMR models (NMR MD) (dark blue), FARFAR2 models which sample the central CG hydrogen bond (FAR CG) (light blue) and FARFAR2 models which sample the central CU hydrogen bond (FAR CU) (red). 6

Supporting Figure 6. Proton chemical shift analysis for four SL2 ensembles: the starting models from the deposited 2L6I NMR (black), the MD simulations starting from those deposited models (NMR MD) (blue), the MD simulations starting from FARFAR2 models which sample the central CG hydrogen bond (FAR CG) (cyan) and the MD simulations starting from FARFAR2 models which sample the central CU hydrogen bond (FAR CU) (red). Measured vs. Predicted ^{1}H shifts are split by proton type. 7

Supporting Figure 7. Time dependent RMSDs of heavy atoms of loop and stem for 10 MD simulations starting from top structures of ten clusters of FARFAR2 models of SL3. Top: Loop residues show

significant rearrangement. Bottom: Stem residues show some propensity for unfolding, noted by high RMSDs........ 8

Supporting Table 2. Cluster analysis for SL3 simulations starting from FARFAR2 predicted structures .. 8

Supporting Figure 8. Top four clusters from SL3 MD simulations. The representative structure is shown in blue with ten evenly spaced frames from the cluster trajectory shown as overlapping transparent structures........ 9

Supporting Figure 9. Closest K^+ ion to binding site for top cluster from SL3 MD simulations. The x-axis shows the atom number of the closest K^+ ion to the binding site, and the y-axis shows the distance to the binding site for that atom. This illustrates that the closest ion exchanges with bulk........ 9

Supporting Figure 10. Comparison to in-cell DMS for SL2 and SL3. RMSF for each residue in each stem loop is averaged over all simulations, with standard deviations reported as error bars (error bars on SL2 plot are present, but too small to be visible for most bases). Background is colored by DMS reactivity, as denoted in reference [24], from 0.0 DMS signal (cyan, least reactive) to 1.0 DMS signal (red, most reactive), with yellow values falling between the two........ 10

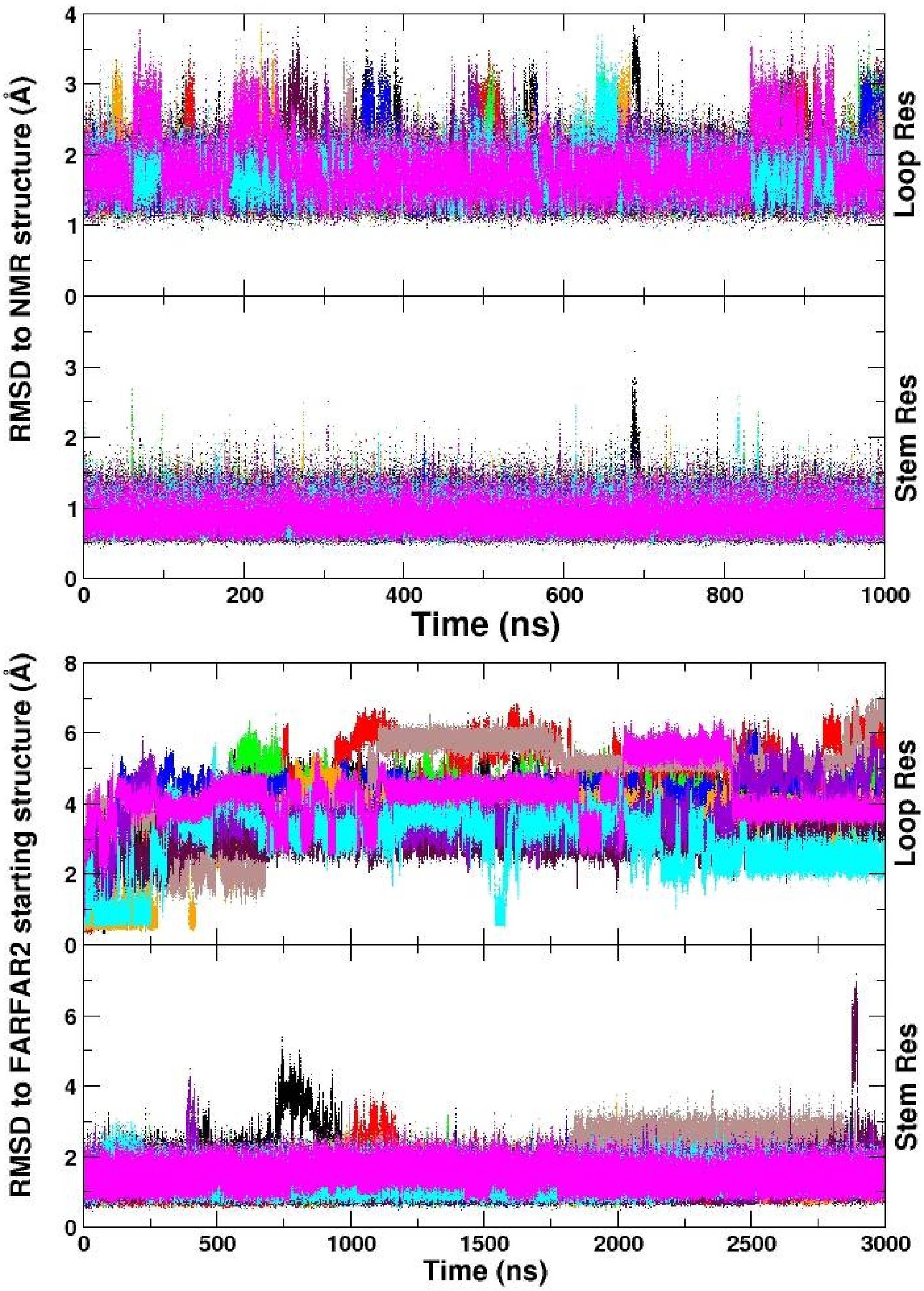

Supporting Figure 1. Time dependent RMSDs to heavy atoms of loop and stem of starting SL2 structures. Top: Simulations starting from NMR ensemble structures. Bottom: Simulations starting from FARFAR2 top structures from clusters 1-10.

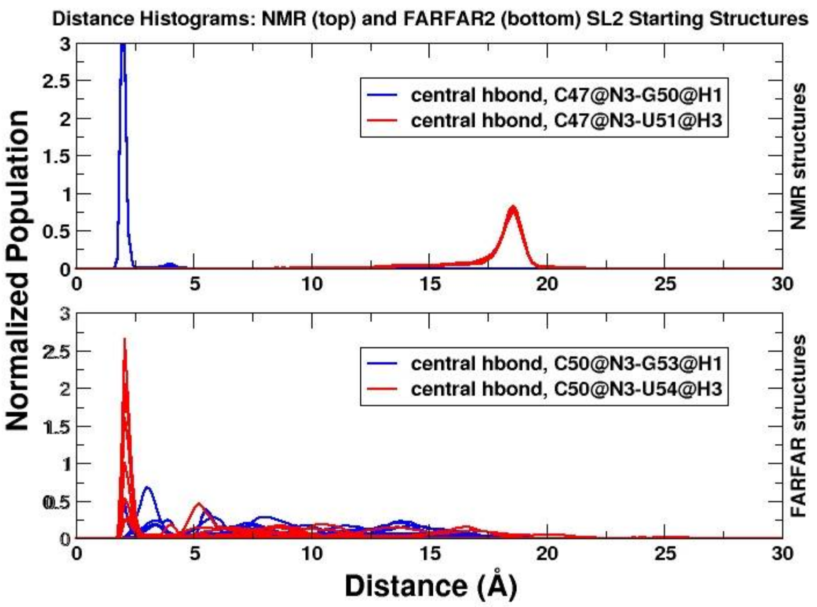

Supporting Figure 2. Histogram of hydrogen bond distance between N3 and H1 atoms of C-G (NMR: C47-G50, FARFAR2: C50-G53, blue) or N3 and H3 atoms of C-U (NMR: C47-U50, FARFAR2: C50-U54, red) measured for SL2 simulations starting from NMR ensemble structures (top) and simulations starting from FARFAR2 top structures from clusters 1-10 (bottom).

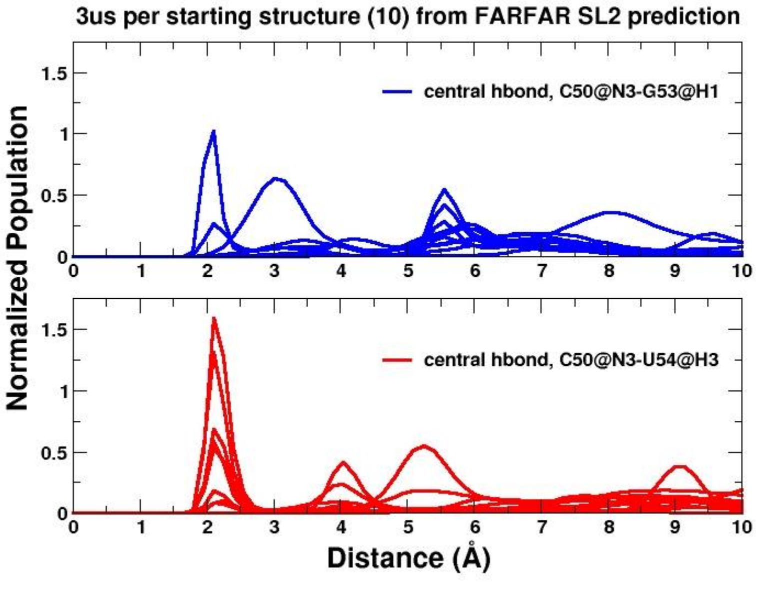

Supporting Figure 3. Close up of histogram of hydrogen bond distance between N3 and H1 atoms of C50-G53 (top, blue) or N3 and H3 atoms of C50-U54 (bottom, red) measured for SL2 simulations starting from FARFAR2 top structures from clusters 1-10. This is a close up of the bottom plot of Supporting Figure 2.

Supporting Table 1. Number of frames and percentage of FARFAR2-SL2 trajectories sampling central CG/CU base pair

Run	Number of frames sampling central CG bp	Percentage of trajectory frames	Number of frames sampling central CU bp	Percentage of trajectory frames
1	132	0.04	63831	21.28
2	15151	5.05	83284	27.76
3	9394	3.13	13671	4.56
4	743	0.25	15929	5.31
5	4	0.00	63530	21.18
6	218	0.07	150849	50.28
7	1024	0.34	259	0.09
8	9519	3.17	649	0.22
9	4	0.00	188171	62.72
10	98008	32.67	21993	7.33

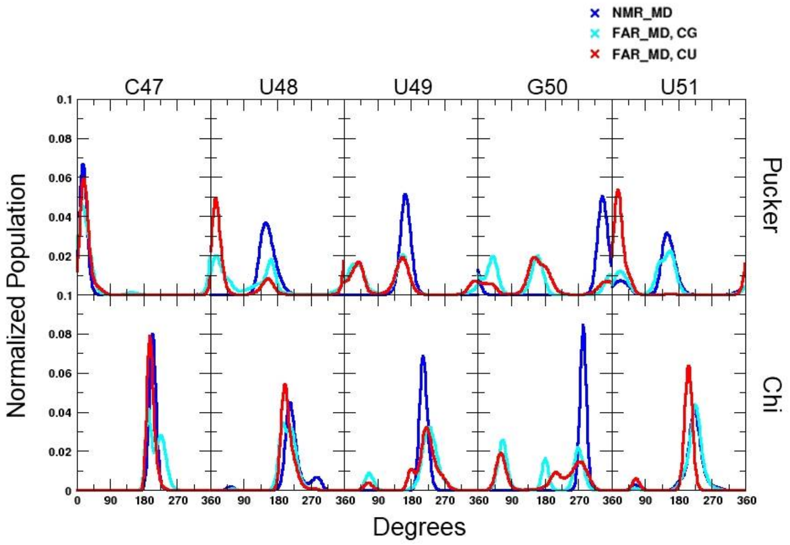

Supporting Figure 4. Pucker and chi distributions for SL2 MD simulations starting from: deposited NMR models (NMR MD) (blue), FARFAR2 models which sample the central CG hydrogen bond (FAR CG) (cyan) and FARFAR2 models which sample the central CU hydrogen bond (FAR CU) (red). SL2 numbering scheme from NMR structure is used here for both NMR and FARFAR2 models.

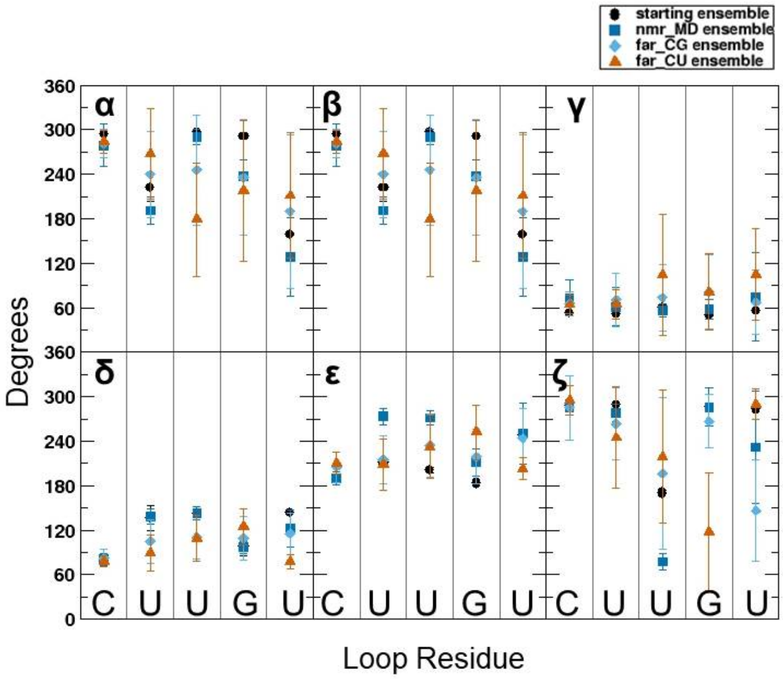

Supporting Figure 5. Backbone dihedral average values for SL2 loop residues for the deposited MD models (black) MD simulations starting from: deposited NMR models (NMR MD) (dark blue), FARFAR2 models which sample the central CG hydrogen bond (FAR CG) (light blue) and FARFAR2 models which sample the central CU hydrogen bond (FAR CU) (red).

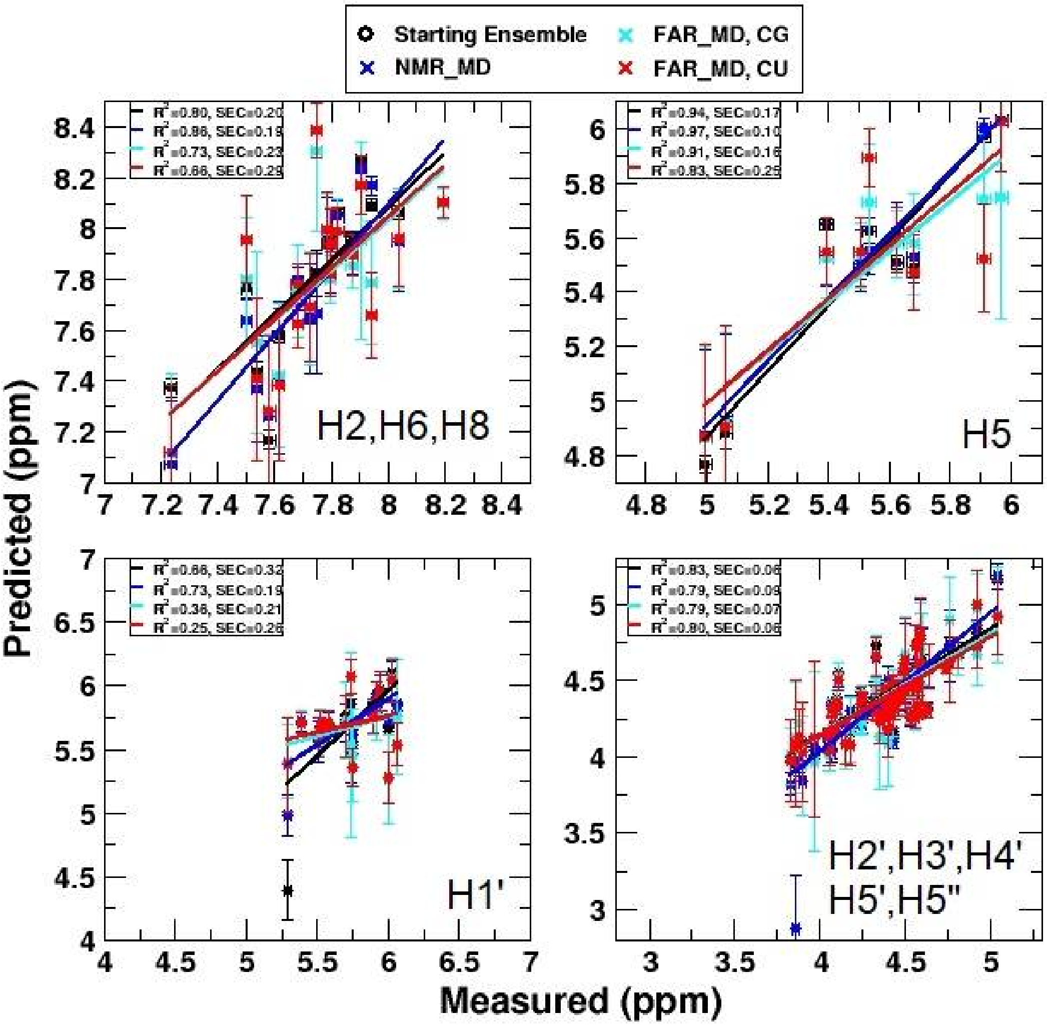

Supporting Figure 6. Proton chemical shift analysis for four SL2 ensembles: the starting models from the deposited 2L6I NMR (black), the MD simulations starting from those deposited models (NMR MD) (blue), the MD simulations starting from FARFAR2 models which sample the central CG hydrogen bond (FAR CG) (cyan) and the MD simulations starting from FARFAR2 models which sample the central CU hydrogen bond (FAR CU) (red). Measured vs. Predicted ^{1}H shifts are split by proton type.

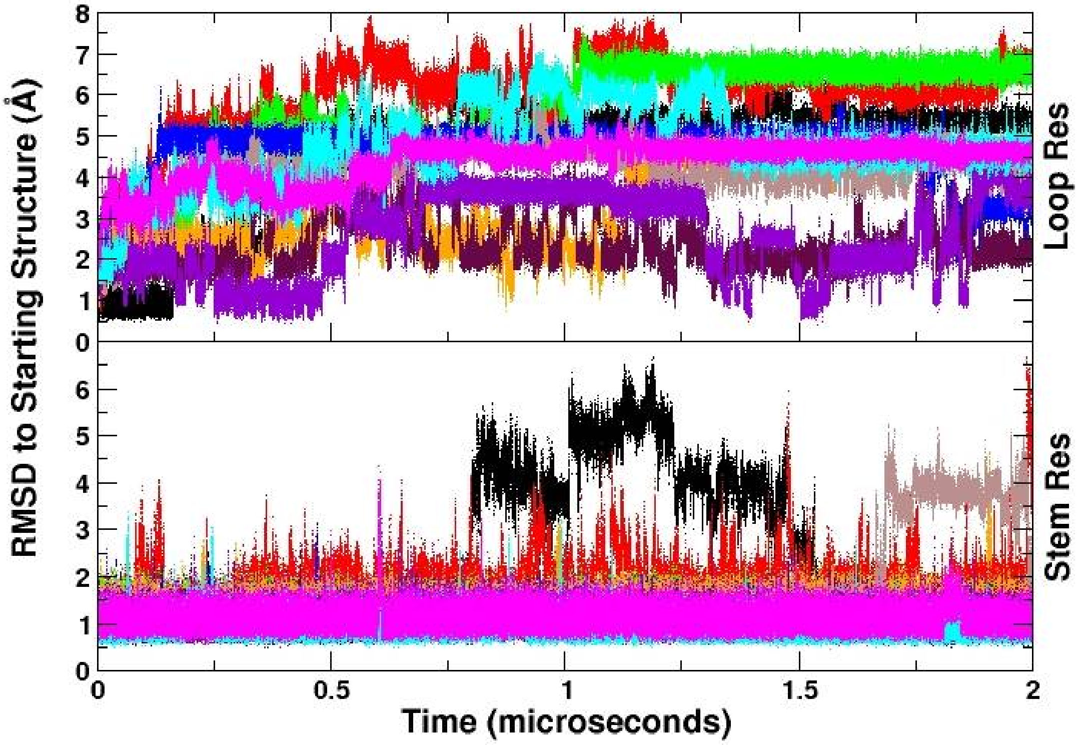

Supporting Figure 7. Time dependent RMSDs of heavy atoms of loop and stem for 10 MD simulations starting from top structures of ten clusters of FARFAR2 models of SL3. Top: Loop residues show significant rearrangement. Bottom: Stem residues show some propensity for unfolding, noted by high RMSDs.

Supporting Table 2. Cluster analysis for SL3 simulations starting from FARFAR2 predicted structures

Cluster	Frames	Fraction	Average distance btwn points (Å)	Stdev	RMSD to representative structure (Å)	Stdev
0	6182	0.31	2.99	0.79	2.36	0.64
1	3596	0.18	2.96	0.84	2.30	0.56
2	2647	0.13	2.42	0.72	2.39	0.91
3	1679	0.08	1.39	0.52	1.27	0.45
4	1081	0.05	3.77	0.85	3.12	0.54
5	1009	0.05	2.03	0.99	1.55	0.72
6	977	0.05	2.68	0.97	2.25	0.74
7	976	0.05	1.58	0.60	1.30	0.45
8	753	0.04	3.08	1.01	2.67	0.59
9	700	0.04	1.27	0.56	1.09	0.41
10	400	0.02	3.74	0.96	2.86	0.94

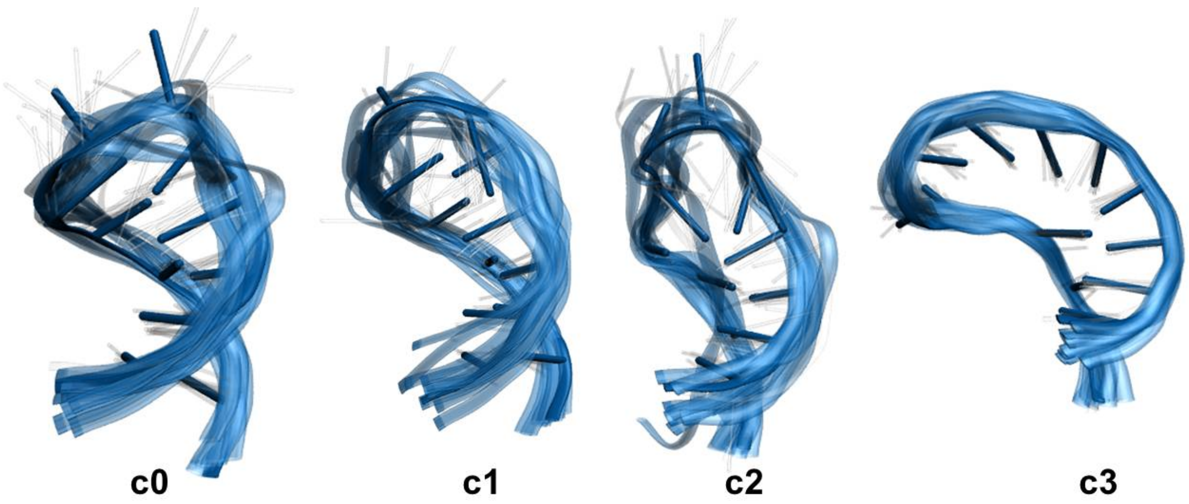

Supporting Figure 8. Top four clusters from SL3 MD simulations. The representative structure is shown in blue with ten evenly spaced frames from the cluster trajectory shown as overlapping transparent structures.

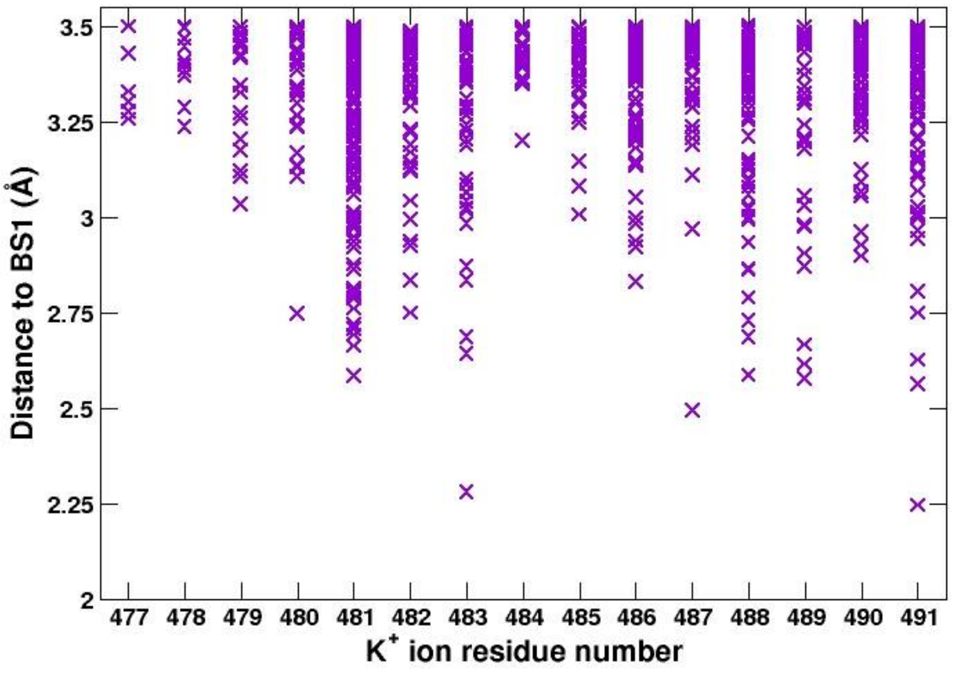

Supporting Figure 9. Closest K+ ion to binding site for top cluster from SL3 MD simulations. The x-axis shows the atom number of the closest K+ ion to the binding site, and the y-axis shows the distance to the binding site for that atom. This illustrates that the closest ion exchanges with bulk.

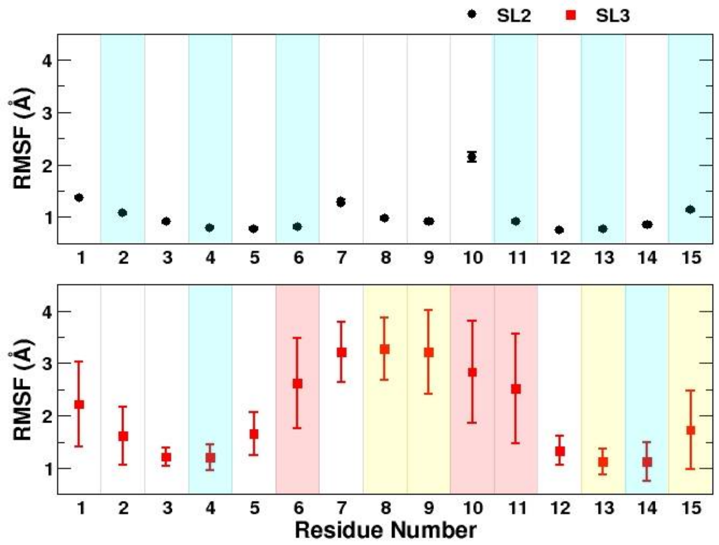

Supporting Figure 10. Comparison to in-cell DMS for SL2 and SL3. RMSF for each residue in each stem loop is averaged over all simulations, with standard deviations reported as error bars (error bars on SL2 plot are present, but too small to be visible for most bases). Background is colored by DMS reactivity, as denoted in reference [24], from 0.0 DMS signal (cyan, least reactive) to 1.0 DMS signal (red, most reactive), with yellow values falling between the two.
